# Scalable Fabrication of 4 nm Silicon Nanopores by Self-Limiting Metal-Assisted Chemical Etching Combined with Optical Process Control

**DOI:** 10.1021/acs.langmuir.6c00975

**Published:** 2026-06-25

**Authors:** Fabio De Ferrari, Alessandro Enrico, Chrysovalantou V. Leva, Shyamprasad N. Raja, Anna Herland, Frank Niklaus, Göran Stemme

**Affiliations:** † Department of Micro and Nanosystems, 166474 KTH Royal Institute of Technology, Malvinas väg 10, Stockholm 100 44, Sweden; ‡ Synthetic Physiology Lab, Department of Civil Engineering and Architecture, 19001 University of Pavia, via Adolfo Ferrata 9, Pavia 27100, Italy; § Division of Nanobiotechnology, SciLifeLab, Department of Protein Science, 7655 KTH Royal Institute of Technology, Tomtebodavägen 23a, Solna 171 65, Sweden; ∥ AIMES - Center for Integrated Medical and Engineering Science, Department of Neuroscience, Karolinska Institute, Stockholm 17177, Sweden

## Abstract

Solid-state nanopores in ultrathin (<20 nm) membranes enable label-free single-molecule sensing, but their adoption as sensors is limited by the lack of scalable manufacturing methods that deliver nanopores with single-nanometer reproducibility. Self-limiting metal-assisted chemical etching (MACE) in silicon-on-insulator (SOI) membranes offers a parallel wet-chemical route for nanopore fabrication, yet prior demonstrations lacked a predictive design rule and required electron microscopy or electrical tests for confirming presence and number of pores. Here, we convert self-limiting MACE into a manufacturing-oriented workflow with optical process control to obtain and verify the formation of 4 nm nanopores in a scalable fashion. We decouple the deposition of 200 ± 10 nm gold (Au) nanoparticles from etching, enabling independent optimization of the two steps. The nanoparticle size allows for particle-per-membrane counting by dark-field optical microscopy, so that deposition can be repeated when counts are below target. We then map etching behavior across Au nanoparticle diameter *d* (10–200 nm) and silicon (Si) device-layer thickness *t* (5–18 nm), finding that *d*/*t* ≥ 0.8 ratio predicts self-limiting MACE behavior, where pore diameter becomes independent of particle size. In this regime, 200 ± 10 nm catalysts yield 4 ± 1 nm pores, corresponding to a reduction of ∼50× in pore diameter and ∼10× in pore-diameter variability compared to the catalyst diameter and related variability. Successful through-membrane pore formation produces undercuts in the buried oxide (typically ∼200–300 nm diameter) beneath each pore, which can be characterized for each membrane by bright-field microscopy and used as a proxy for the otherwise optically invisible 4 nm pores. Together, the predictive *d*/*t* framework and two-stage optical verification establish a scalable wet-chemical route to fabricate nanopores for biomolecular sensing and related nanofluidic devices.

## Graphical abstract

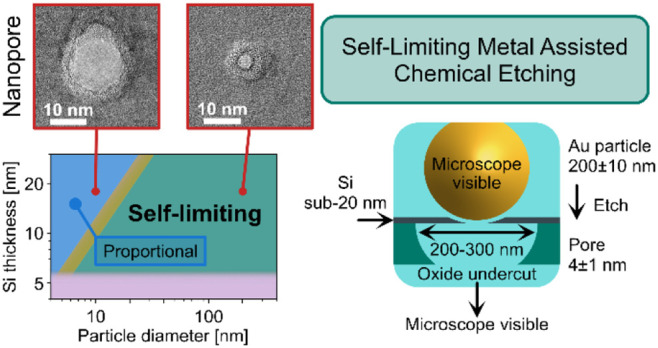

## 1 Introduction

Solid-state nanopores in ultrathin (<20 nm) membranes have emerged as powerful platforms for label-free single-molecule sensing in genomics, proteomics, and molecular diagnostics.^1–6^ However, the broad adoption of solid-state nanopores requires scalable manufacturing methods that deliver large numbers of nanopores with single-digit nanometer diameters across large areas and large numbers of devices, which is currently an open challenge. In realizing sub-10 nm pores, precise dimensional control of the fabricated pore is essential to minimize electrical noise, ensure reproducible molecular transport, and achieve device-to-device uniformity.^7–15^ Furthermore, rapid and nondestructive verification of pore formation is highly desirable, allowing for efficient in-line process control. Despite substantial progress, existing nanopore fabrication strategies rarely satisfy resolution, throughput, and manufacturability simultaneously. Serial nanopore fabrication techniques such as transmission electron microscopy (TEM) drilling/sculpting,^8^ focused ion beam (FIB) milling,^16^ and controlled dielectric breakdown (CBD)^17–20^ can form sub-10 nm nanopores; however, these approaches are intrinsically throughput-limited, typically requiring feedback control in the fabrication of each individual pore, and exhibiting substantial pore-to-pore variability that necessitates postfabrication characterization and selection of each individual pore. These limitations hinder the viability of such techniques for high-volume and large scale nanopore production.^1,3,4,6^ Lithography-based approaches to fabricate nanopores improve throughput yet remain limited to the realization of pores with diameters of ∼10–20 nm due to fundamental optical and shot-noise constraints of existing lithographic processes.^21,22^ Thus, new large-scale manufacturing techniques are needed to realize precisely defined sub-10 nm diameter pores.

Metal-assisted chemical etching (MACE) of silicon (Si) is an attractive manufacturing process for scalable nanopore fabrication, as it is inherently parallel and compatible with wet-chemical batch processing.^23–25^ In MACE, noble-metal catalysts such as gold (Au) nanoparticles locally oxidize and dissolve the Si in the presence of a liquid hydrofluoric acid (HF) and hydrogen peroxide (H_2_O_2_) solution, thereby resulting in the formation of pores and nanostructures.^26^ Conventional MACE typically yields pores with diameters that are comparable to the dimensions of the nanoparticle,^27,28^ implying that the formation of sub-5 nm pores requires the use of sub-5 nm nanoparticles. We recently reported that MACE in an ultrathin (12 nm thick) Si device layer of a silicon-on-insulator (SOI) substrate can be self-limiting, where the Si etching beneath 10 and 40 nm diameter Au nanoparticles stops at the interface between the Si device layer and the buried oxide (BOX) layer of the SOI substrate.^29^ When using this process to form pores in a suspended Si device layer with a supporting BOX layer, the diameters of the resulting pores (sub-5 nm) are decoupled from the diameters of the used Au nanoparticles in the MACE process. In our previous work, we demonstrated nanopore formation by scanning electron microscopy (SEM), TEM, and ionic-current measurements, focusing primarily on the proof-of-concept of the proposed approach using 10 and 40 nm diameter Au particles. However, a predictive framework for how the Si device-layer thickness, Au nanoparticle size, and etching conditions influence the size of the resulting nanopores does not exist today. Furthermore, the 10 and 40 nm nanoparticles used in the previous study cannot be readily detected with conventional optical microscopy. If larger nanoparticles could be used in the proposed MACE process to form sub-5 nm diameter pores, it is feasible to employ optical microscopy to image these particles and enable optical process control of nanoparticle placement and pore formation before and after the MACE process.

Here, we address these gaps by developing a predictive experimental framework for self-limiting MACE (sMACE) and demonstrating the use of 200 nm diameter particles to fabricate 4 nm diameter pores under optical process control. We systematically vary the Si device layer thickness (5–18 nm) and the Au nanoparticle diameter (10–200 nm) and quantify the resulting pore diameters using TEM. The sMACE process with 200 nm particles yielded 4 nm pores with 1 nm variability. Critically, the use of these large particles enables optical microscopy-based process control at two stages of the fabrication process: dark-field imaging verifies nanoparticle deposition and location before etching, and bright-field imaging detects characteristic undercuts in the BOX layer (200–300 nm) surrounding a formed nanopore, which can be used as an optical pass/fail signature to confirm complete pore formation. This optical signature distinguishes single- from multiple-pore membranes directly at the device level, which conductance alone cannot unambiguously resolve. Unlike serial nanopore-fabrication methods such as TEM drilling and CBD, which rely on per-pore feedback during fabrication, sMACE is intrinsically parallel and requires only wet-chemistry and microscopy. To validate sMACE pores for sensing, we characterize them electrically and via DNA translocation and benchmark their noise performance against two in-house CBD reference devices with comparable open-pore conductance, measured on the same setup. Together, these results establish sMACE as a scalable, optically verifiable route to 4 nm nanopore fabrication compatible with automated machine vision.

## 2 Results and Discussion

To develop a nanopore fabrication process compatible with optical verification, we fabricated suspended ultrathin (<20 nm) Si membranes from SOI wafers (70 nm Si device layer, 145 nm BOX layer, and 400 μm Si handle layer), with the BOX layer serving as mechanical support. To obtain the target device layer thickness, we thermally oxidized the Si device layer of the SOI substrate from its initial thickness (*t*) of 70 nm down to *t* = 5, 12, or 18 nm and subsequently protected the front side (i.e., the device layer) with blue tape while removing the thermal oxide layer only from the backside by HF wet etching. We then deposited and patterned a 270 nm thick SiN_*x*_ passivation layer on both sides of the substrate. Optical lithography and dry etching were used to pattern circular openings of 8 μm diameter in the SiN_*x*_ layer on top of the Si device layer (front side of the SOI substrate), selectively exposing the surface of the Si device layer at the bottom of the circular openings (Figure 1A). We formed square openings in the SiN_*x*_ layer on top of the Si handle layer (backside of the SOI substrate) and anisotropically KOH etched the Si handle layer to create freestanding membranes of ∼50 μm × 50 μm consisting of the SiN_*x*_ passivation, thermal oxide, Si device layer, and the BOX layer. During KOH etching, we used the BOX layer as an etch stop (see Methods for details). To define the location for nanopore channel formation, a single 8 μm diameter circular opening in the SiN_*x*_ layer was placed at the center of each membrane. Additionally, to evaluate the particle deposition process, we designed four arrays of 6 × 6 openings (each 8 μm in diameter) in the SiN_*x*_ layer at distinct locations beside the membrane of each device serving as evaluation points (Figure 1B, top). The suspended Si/BOX membrane area and the SiN_*x*_ openings were readily identifiable in the optical microscope (Figure 1B, bottom). Each device consists of a 2.5 mm × 2.5 mm area with a single membrane at its center. We performed all experiments on substrates of 2.5 mm × 7.5 mm (containing 3 devices) or 5 mm × 7.5 mm (containing 6 devices).

**Figure 1:**
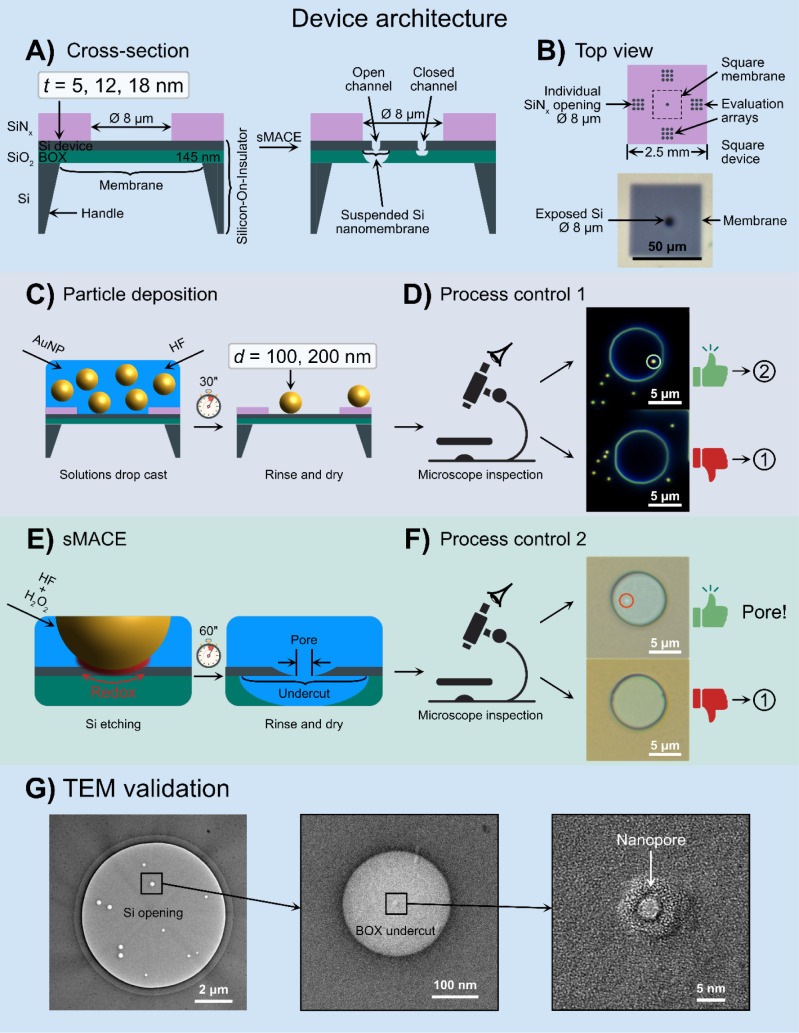
Nanopore fabrication by sMACE with optical process control. (A) Cross-section of SOI membrane (*t* = 5, 12, or 18 nm Si device layer, 145 nm BOX). Open channel requires complete BOX removal beneath the pore. (B) Top: device layout with central membrane opening and four 6 × 6 evaluation arrays. Bottom: optical image of membrane (scale bar: 50 μm). (C) Au nanoparticle deposition by drop-casting with HF (*d* = 100–200 nm for optical detection). (D) Pre-MACE process control checkpoint (stage 1): dark-field detection of deposited particles. (E) sMACE (60 s) forms nanopores and BOX undercuts. (F) Post-MACE process control checkpoint (stage 2): bright-field detection of undercuts confirms pore formation. (G) TEM validation at increasing magnification: 8 μm opening with multiple undercuts (left, scale bar: 2 μm), single undercut (center, scale bar: 100 nm), and ∼4 nm nanopore (right, scale bar: 5 nm).

To fabricate nanopores suitable for biomolecular sensing, we developed a process combining self-limiting MACE with optical process control using Au nanoparticles. Optical process control enables a two-stage verification scheme: first, the Au particles deposited on the substrate surface, specifically within the circular SiN_*x*_ opening, can be counted before performing the MACE process (Figure 1C, D). Second, nanopore formation can be confirmed through imaging of an etched undercut in the BOX layer surrounding the pore opening in the Si layer after the MACE process (Figure 1E, F). Since biomolecular sensing typically requires individual nanopores or small arrays of nanopores to optimize signal-to-noise ratios,^3^ we targeted single-particle deposition within each of the 8 μm openings in the SiN_*x*_ layer on the membrane. We systematically evaluated Au nanoparticles with diameters of *d* = 10, 15, 20, 40, 100, and 200 nm and observed that particles of 100 and 200 nm are readily visible using standard optical microscopy (Figure 1C).

In our experiments, we assessed the number of deposited Au particles by epi-dark-field optical imaging as a first process control step. Devices displaying the desired particle count within the exposed Si evaluation areas proceeded to etching, while those with low counts could be reused by repeating the deposition step (Figure 1D). To form functional pores, the MACE etching solution was drop-cast onto the substrate. The self-limiting nature of the sMACE process ensured that once a pore formed beneath an Au particle, Si etching stopped and the particle detached from the surface, leaving an open pore. The presence of HF in the etchant resulted in undercuts in the BOX layer surrounding each open pore (Figure 1E). To form a functional open channel through the entire Si/BOX membrane, the undercut in the BOX layer needed to be at least 150 nm in diameter for the 145 nm thick BOX layer (Figures 1A, S2). Conveniently, undercuts of this size were visible by epi-bright-field optical microscopy, enabling the second process control stage where we could immediately confirm pore formation after the MACE process (Figure 1F). Devices without successfully formed pores could be reused for another nanoparticle deposition and MACE process cycle.

To validate nanopore dimensions, we performed TEM imaging on selected samples. The 8 μm SiN_*x*_ openings on the membranes effectively localized pore formation within a well-defined region, as shown in the TEM overview where individual undercuts appeared as bright spots (Figure 1G, left). The undercuts, centered around each pore, facilitated the immediate identification of pore location during TEM imaging (Figure 1G, center). High-magnification TEM confirmed the formation of pores with a diameter of 4 nm (Figure 1G, right), with detailed size distributions reported below and measurement protocols described in the Methods section.

### 2.1 Self-Limiting MACE Regime

To establish predictive control over nanopore formation, we investigated the parameter space under which MACE becomes self-limiting, defined here as the regime where Si etching terminates upon reaching the Si-BOX interface, resulting in pore dimensions independent of particle size. We selected MACE solution compositions with molar ratios of ρ = [HF]/([HF]+[H_2_O_2_]) > 0.7, which are reported to produce straight cylindrical pores in Si.^23,26^ Higher HF concentrations also accelerate BOX etching once a pore is formed, which is essential for forming fully open undercuts beneath each pore. With typical MACE etch rates in the μm/min regime^25,30^ and Si device layer thicknesses of ≤18 nm, we selected 60 s as a practical minimum for consistent manual solution handling. Analysis of the resulting pore geometries revealed that the particle diameter-to-Si device thickness ratio (d/t) reliably predicted whether MACE operated in one of three regimes, here termed proportional, self-limiting, and stochastic. In the proportional regime (*d*/*t* < 0.8), the pore diameter scales with the nanoparticle diameter, consistent with conventional MACE behavior. In the self-limiting regime (*d*/*t* ≥ 0.8), etching terminates at the Si–BOX interface, and the resulting pore diameter becomes independent of particle size, yielding 4 nm pores regardless of the catalyst dimensions. Within the self-limiting regime, for *d*/*t* values between approximately 0.8 and 1.1, nanoparticles stochastically remain at the etch site after pore formation, preventing direct TEM measurement of pore diameter despite undercuts confirming that pores have formed. We refer to this subregime as the stochastic detachment region; reported pore diameters are only for samples in which the nanoparticle detached after etching. At d/t values above 1.1, nanoparticles consistently detach, enabling TEM verification of pore dimensions.

#### 2.1.1 Critical *d*/*t* Threshold for Self-Limiting MACE Behavior

##### 2.1.1.1 Self-Limiting Regime (*d*/*t* ≥0.8)

To evaluate if optically visible particles (*d* ≥ 100 nm) can form 4 nm pores via self-limiting MACE, we characterized pore formation using Au nanoparticles of *d* = 15, 20, 40, and 200 nm in *t* = 18 nm thick Si device layers on top of the BOX, corresponding to *d*/*t* = 0.8, 1.1, 2.2, and 11.1, respectively. We used a MACE solution with volume ratio DI:HF:H_2_O_2_ = 160:320:1, corresponding to ρ = 0.998. Unlike conventional MACE, where pore diameter typically scales proportionally with particle diameter,^28^ all evaluated particle sizes in the self-limiting regime formed pores with diameters of 4.8 ± 0.9 nm (*N* = 27, Figure S1). This particle size-independent pore formation behavior represents a fundamental departure from the pores of 11.5 nm produced by Au particles of *d* = 10 nm (*d*/*t* = 0.6), consistent with proportional MACE behavior (Figure 2A, Table 1; discussed in the next section). Figure 2C, D show pores formed by *d* = 40 and 200 nm particles, respectively. Most notably, 200 nm particles with ±10 nm manufacturer variability produced pores with only ± 1 nm spread, a 10-fold improvement in dimensional control (Table 1).

**Table 1:** Summary of MACE Regimes and Pore Diameters as a Function of Particle Diameter and Si Device Layer Thickness^a^

t [nm]	*d* [nm]	*d*/*t*	Regime	Pore diameter [nm]	*N*
18	10 ± 2	0.6	Proportional	11.5 ± 1.9	11
18	15 ± 2	0.8	Stochastic	5.3 ± 1.2	8
18	20 ± 2	1.1	Stochastic	4.3 ± 0.7	4
18	40 ± 2	2.2	Self-limiting	4.8 ± 0.4	9
18	200 ± 10	11.1	Self-limiting	4.6 ± 1.2	6
12	40 ± 2	3.3	Self-limiting	4.4 ± 1.3	19
12	100 (+10/-2)	8.3	Self-limiting	4.1 ± 1.0	5
12	200 ± 10	16.7	Self-limiting	4.5 ± 1.0	6

a *t* (Si device layer thickness), *d* (Au nanoparticle diameter), *d*/*t* (particle-to-thickness ratio), pore diameter (mean pore diameter ± SD), *N* (number of TEM-measured pores). Particle diameters as reported by the manufacturer (Nanopartz Inc.).

**Figure 2:**
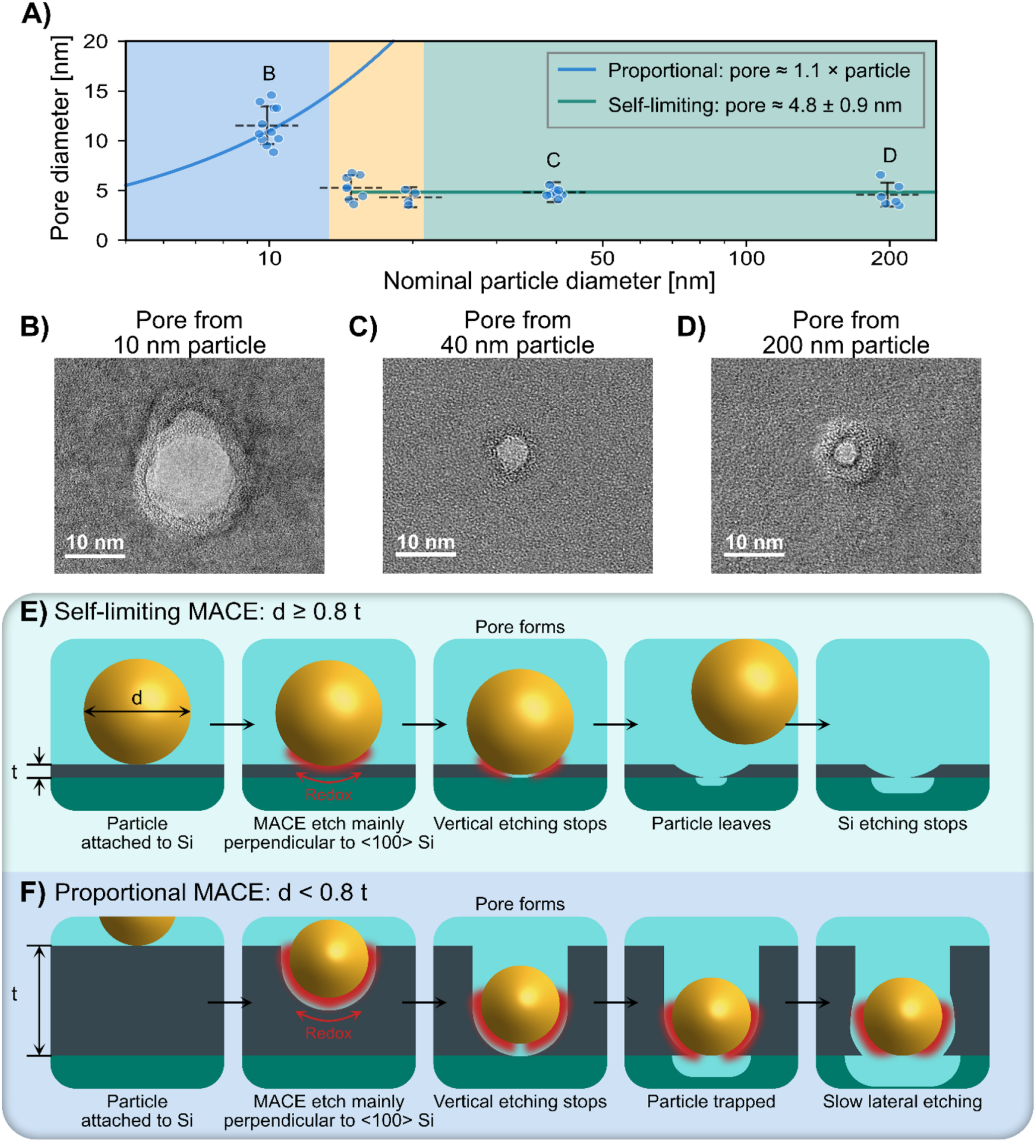
Self-limiting MACE behavior for *d*/*t* ≥ 0.8. (A) Pore diameter vs Au nanoparticle diameter for *t* = 18 nm. Background shading: proportional (blue, *d*/*t* < 0.8), stochastic detachment (yellow, 0.8 ≤ *d*/*t* ≤ 1.1), and reliable detachment (green, *d*/*t* > 1.1). Blue line: proportional scaling (1.1× particle diameter). Green line: self-limiting mean (4.8 ± 0.9 nm). Each point is one pore; per-condition statistics in Table 1. (B-D) TEM images of pores formed by (B) 10 nm, (C) 40 nm, and (D) 200 nm particles. Scale bars: 10 nm. (E) sMACE schematic (*d*/*t* ≥ 0.8): particle detaches after reaching the Si-BOX interface and etching stops. (F) Proportional MACE schematic (*d*/*t* < 0.8): particle trapped in pore, lateral etching continues.

From these observations, we empirically defined the ratio between the particle diameter and the Si device layer thickness *d*/*t* ≥ 0.8 as the condition at which MACE becomes self-limiting. For fabricating isolated 4 nm diameter pores, choosing particles as large as possible within this regime proved highly beneficial, enabling the integration of fully optical process control into our fabrication process.

The MACE etching process becomes self-limiting in this SOI system through a sequence of particle-interface interactions. The Au nanoparticle is initially held in close contact with the Si surface by van der Waals forces.^31–33^ Si at the Au–Si interface is removed by the redox reaction. The Au catalyst injects holes in the Si through H_2_O_2_ reduction and locally oxidizes the Si substrate. The excess HF in solution ensures that any SiO_2_ formed is immediately dissolved, sustaining continuous Si removal. The etching proceeds anisotropically, perpendicular to the <100> Si crystalline plane (Figure 2E). When the Au particle reaches the Si-BOX interface, directly below the particle no more Si is available for etching, and the underlying BOX starts being etched isotropically. Where TEM imaging revealed open pores without Au particles, pore diameters were approximately 4 nm, suggesting that the Au particle detaches from the surface once the pore reaches this critical dimension, thereby terminating Si etching. Undercut etching of the BOX continues after Au particle detachment, creating the optically visible undercut features described in Figure 1F, G.

##### 2.1.1.2 Proportional Regime (*d*/*t* <0.8)

At low particle diameter to Si membrane thickness *d*/*t* ratios, MACE followed the conventional scaling relationship, in which the diameter of the resulting nanopore increases proportionally with the particle size. Au nanoparticles of *d* = 10 nm in *t* = 18 nm thick Si membranes (*d*/*t* = 0.6) formed pores of 11.5 nm diameter (Figure 2A, B, Table 1). The pore-to-particle size ratio of 1.1× matches the ratio we computed from the data of Liu et al. for MACE in bulk Si,^28^ indicating that proportional etching behavior extends from bulk Si substrates to the device layer of SOIs.

In the proportional regime, Au particles fully penetrate the Si device layer and continue etching after reaching the Si-BOX interface until all surrounding Si is removed (Figure 2F). The particle detaches only after etching completely through the Si device layer, creating pores with diameter determined by the particle cross-section. This result provides additional context for interpreting our previous observation that 10 nm particles in a *t* = 12 nm thick Si device layer (*d*/*t* = 0.8) remained trapped after forming sub-5 nm pores.^29^ By evaluating the MACE process using the same particle size in a thicker Si device layer (*t* = 18 nm, *d*/*t* = 0.6), we observed that the Si-BOX interface does not prevent full particle penetration at sufficiently low d/t ratios, confirming that pore formation in the proportional MACE regime can occur in the Si device layer of a SOI substrate. Together with our characterization results in the sMACE regime, these observations establish that the Au particle size relative to the Si device layer thickness is a governing parameter that determines MACE behavior in a Si device layer placed on top of a BOX layer.

#### 2.1.2 Role of Etch Solution in MACE

To optimize the MACE chemistry, we pursued two objectives: leveraging the sMACE regime to form 4 nm pores and etching BOX undercuts large enough to create open channels through the entire membrane stack. Previous reports established that solution compositions with molar ratios of ρ > 0.7 produce vertical cylindrical pores in Si,^23,34^ providing a starting point for our experiments. We began with a volumetric ratio of DI:HF:H_2_O_2_ = 20:40:1 (ρ = 0.985, H_2_O_2_ concentration = 155 mM), where we expected self-limiting MACE behavior to dominate. Under these conditions, MACE exhibited the anticipated self-limiting characteristics, but the Au particles remained in contact with the Si surface while displaying lateral motion along the Si substrate surface. SEM imaging revealed that this chemistry produced elongated slits across the Si substrate surface with sub-10 nm widths and lengths ranging from nanometers to several micrometers (Figure 3B). These results revealed that while MACE remained self-limiting under these conditions, particle mobility enabled continued lateral etching rather than particle detachment.

**Figure 3:**
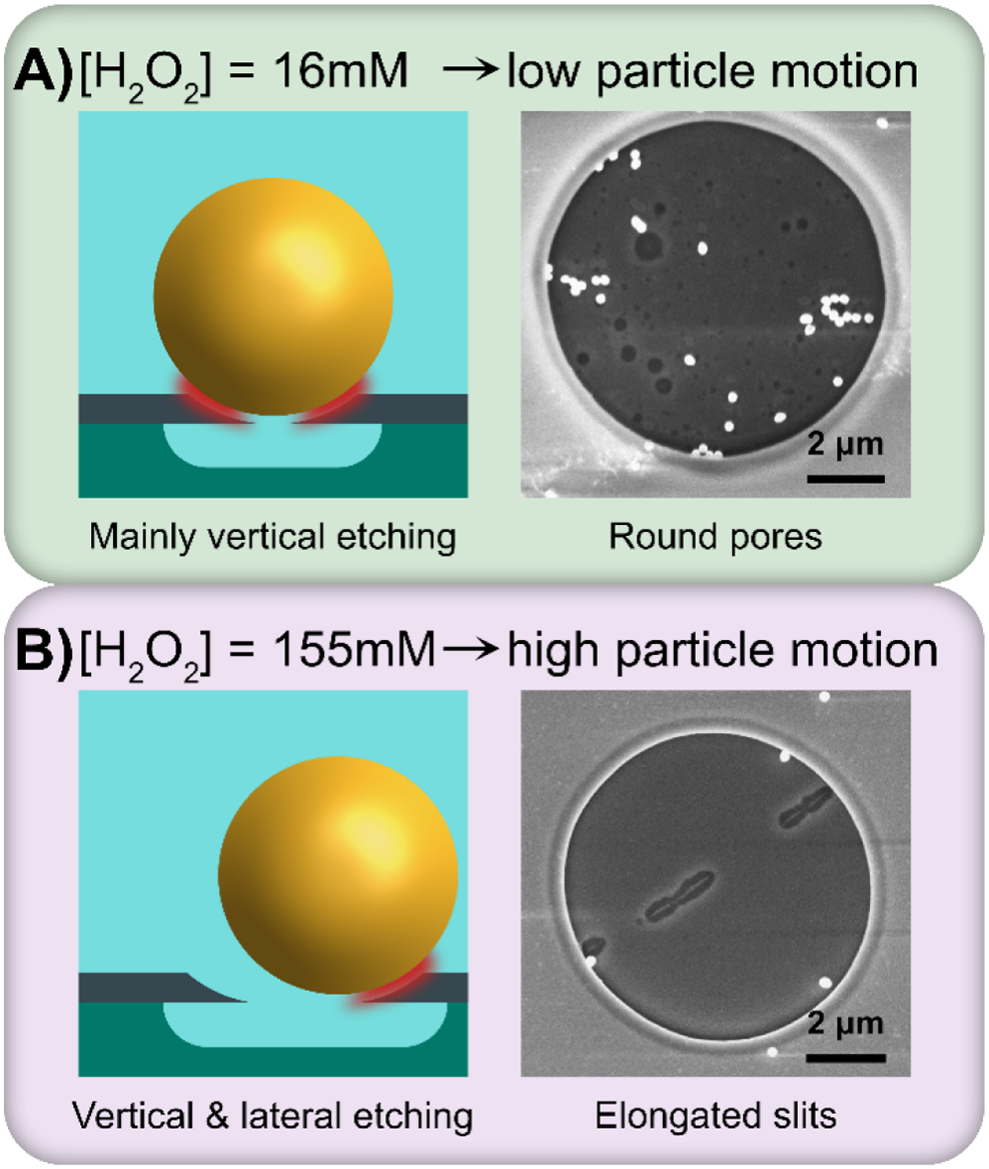
H_2_O_2_ concentration controls pore morphology. (A) Low H_2_O_2_ (16 mM) produces round pores. (B) Higher H_2_O_2_ concentrations (e.g., 155 mM) cause lateral particle motion, producing elongated slits. Both panels: *t* = 18 nm, *d* = 200 nm (*d*/*t* = 11.1), scale bars 2 μm. Left: schematics of particle motion during etching. Right: corresponding SEM images.

Reducing H_2_O_2_ concentration is known to reduce catalyst motion in MACE;^33^ therefore, to suppress lateral Au particle motion, we decreased the H_2_O_2_ concentration while maintaining constant HF and DI concentrations. Evaluating volumetric ratios of DI:HF:H_2_O_2_ = 40:80:1, 80:160:1, and 160:320:1, we found that only the latter consistently resulted in round pores without lateral Au nanoparticle displacement. This optimized composition corresponds to ρ = 0.998 and H_2_O_2_ concentration of 16 mM, representing a 10-fold reduction in H_2_O_2_ concentration compared to the initial DI:HF:H_2_O_2_ = 20:40:1 composition (Figure 3A).

#### 2.1.3 Role of Si Device-Layer Thickness

In nanopore biosensors, reduced membrane thickness directly enhances spatial resolution along the analyte translocation axis, making ultrathin Si device layers desirable.^35–37^ Having established self-limiting behavior in a *t* = 18 nm thick Si device layer supported by a BOX layer, we systematically investigated whether thinner device layers could maintain sMACE behavior. For all experiments in this section, we used the optimized MACE solution composition (DI:HF:H_2_O_2_ = 160:320:1, ρ = 0.998), ensuring that the chemical conditions remained constant while the Si device layer thickness varied.

Building on our previous report of 3.5 ± 1.7 nm (*N* = 3) from *d* = 40 nm particles in *t* = 12 nm Si (*d*/*t* = 3.3),^29^ we replicated this result with 40 nm particles and extended it to 100 and 200 nm (Table 1). Across all particle sizes, the pore diameter was 4.3 ± 1.1 nm (*N* = 30, Figures 4A, S1), confirming that sMACE behavior persists in thinner Si device layers. The improved uniformity compared to our previous report (±1.1 vs ± 1.7 nm)^29^ is consistent with the reduced lateral particle motion achieved by lowering H_2_O_2_ concentration. Representative TEM images show the characteristic undercut morphology for pores formed from 40 nm particles (Figure 4B). The circular BOX undercut diameter of approximately 200 nm surrounding the pore reflects isotropic HF etching and remains consistent across samples. In some cases, tracks extending from the pore indicate lateral motion of the Au particle during MACE (Figure 4C). Despite this lateral particle displacement, the circular BOX undercut uniformly surrounding the pore confirms that HF etching proceeded from a round pore, demonstrating that self-limiting MACE behavior persisted even when particles exhibited some surface mobility before detachment.

**Figure 4:**
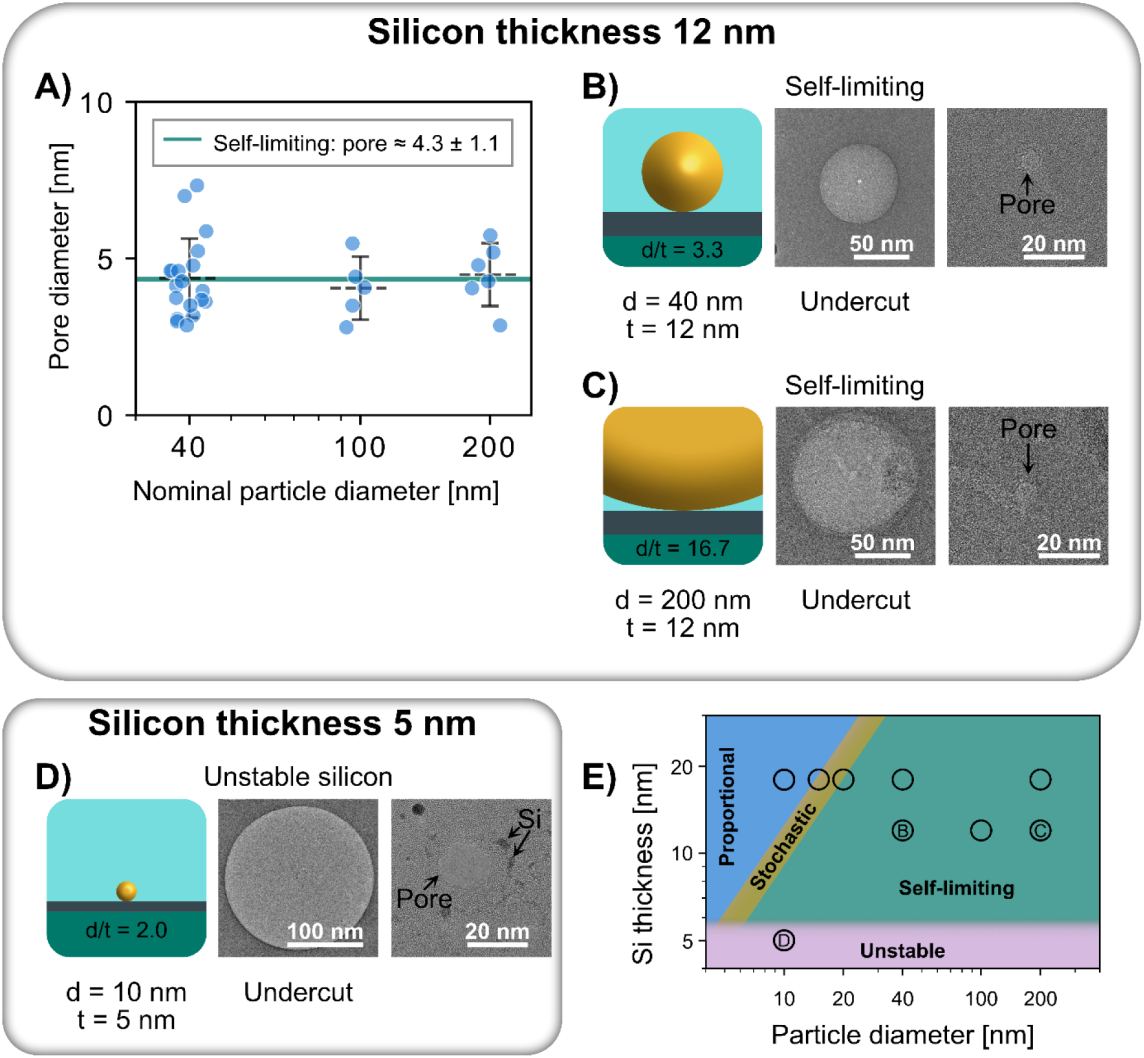
Si device layer thickness scaling. (A) Pore diameter vs Au nanoparticle diameter for *t* = 12 nm. Green line: self-limiting mean (4.3 ± 1.1 nm, *N* = 30). Per-condition statistics in Table 1. (B, C) Schematics (left) and TEM images of undercuts (middle, scale bars: 50 nm) and pores (right, scale bars: 20 nm) for (B) *d* = 40 nm (*d*/*t* = 3.3) and (C) *d* = 200 nm (*d*/*t* = 16.7). (D) Unstable regime: *d* = 10 nm on *t* = 5 nm (*d*/*t* = 2.0). Schematic (left). Membrane fragmentation (center, scale bar: 100 nm) and irregular pore >20 nm (right, scale bar: 20 nm). (E) Phase diagram mapping MACE regimes as a function of particle diameter and Si device layer thickness. Circles: experimentally characterized conditions. Letters in circles correspond to panels in this figure.

To establish the smallest Si device layer thickness that is viable as a membrane material, we performed sMACE on *t* = 5 nm thick Si using *d* = 10 nm Au nanoparticles (*d*/*t* = 2.0). TEM imaging revealed resulting pores exceeding 20 nm in diameter with irregular, noncircular geometries and rough edges (Figure 4D). These pore dimensions were larger than the size of the used particles and substantially larger than the 4 nm diameter pores that we observed forming in thicker Si device layers under similar *d*/*t* conditions. Additionally, Si fragments were visible near the pores, indicating membrane material detachment from the pore perimeter (Figure 4D, right). These observations indicate that 12 nm thick Si membranes support controlled sMACE while 5 nm thick membranes do not. Although we did not systematically determine the exact minimum viable thickness of the Si membrane, these results establish Si membrane thicknesses of *t* ≥ 12 nm as a conservative design guideline for a reliable sMACE process.

Figure 4E summarizes our findings in a phase diagram that maps MACE process regimes (with pores evaluated using TEM) as a function of Au nanoparticle diameter and Si device layer thickness, as reported in this work. The diagram displays four distinct MACE process regimes. In the proportional MACE regime (*d*/*t* < 0.8, blue shading), pore diameter scales with particle size following the 1.1× relationship observed in conventional MACE. The self-limiting MACE regime (*d*/*t* ≥0.8) produces pores of 4 nm diameter independent of particle size. Within this regime, two subregions are distinguished based on particle detachment behavior. In the stochastic detachment region (0.8 ≤ *d*/*t* ≤ 1.1, yellow shading), particle retention is variable: some particles detach after forming 4 nm pores while others remain trapped in the Si device layer, preventing direct TEM measurement of pore diameter despite undercuts confirming that pores have formed. In the reliable detachment region (*d*/*t* > 1.1, green shading), particles consistently detach, enabling both TEM verification of pore dimensions and the use of optically visible Au nanoparticles for process control. To apply optical process control, using large Au particles while maintaining *d*/*t* > 1.1 ensures reliable particle detachment within the self-limiting MACE regime. Finally, the regime in which the Si membrane surrounding the pore becomes mechanically unstable (purple shading) indicates conditions where the Si device layer stability precludes the formation of nanopores with predictable sizes, regardless of particle size. Taken together, this phase diagram provides a predictive framework for selecting Au nanoparticle sizes and Si device layer thicknesses, with pooled mean pore diameter across both thicknesses yielding 4.6 ± 1.1 nm (*N* = 57, Figure S1).

### 2.2 Optical Process Control

For scaling nanopore formation using sMACE from laboratory demonstrations to manufacturing-compatible processes it is essential to control the number of nanopores formed in each membrane device, with a single nanopore per membrane being the gold standard. By leveraging 200 nm Au nanoparticles visible in standard optical microscopes and the understanding of the process parameters resulting in sMACE behavior established in this work, we implemented a nanopore fabrication protocol with integrated two-stage optical process control. The first stage monitors the number of particles deposited on the Si membrane surface prior to sMACE (Section 2.2.1) and the second stage monitors the number of pores that have been formed in the Si membrane after the sMACE process (Section 2.2.2). For implementing this protocol, we chose 200 nm Au particles because they can be optically detected in a reliable way while maintaining practical nanoparticle stock solution concentrations. Among the commercially available Au nanoparticle sizes, smaller particles (100 nm) approach the detection limit of standard microscopy, while larger particles (500 nm) require multiple depositions or larger exposure areas due to lower stock concentrations. To demonstrate the nanopore fabrication protocol, we developed automated image analysis algorithms for particle detection and counting (see Methods), providing a foundation for potential integration with automated inspection systems.

#### 2.2.1 Pre-MACE Particle Verification

To confirm the number of Au nanoparticles deposited prior to performing the sMACE process, we imaged each membrane device using epi-dark-field optical microscopy. The deposited 200 nm Au nanoparticles appeared as bright spots against the dark Si background within the 8 μm diameter openings in the SiN_*x*_ layer (Figure 5A, left panel). We used the OpenCV Hough Circle detection algorithm to locate the evaluation openings in the SiN_*x*_ layer beside the membrane area (evaluation points/arrays in Figure 1B) and count the number of Au particles within each opening (Figure 5A, right panel). This provides the first decision point in our protocol: if the particle deposition density is lower than desired, Au particle deposition can be repeated; if the particle deposition density is too high, the Au particles can be removed by wet or dry etching before restarting the deposition. To demonstrate predictable control over particle density, we performed sequential Au particle depositions on the same substrate, adding Au particles 1, 2, 3, and 4 times using an undiluted Au nanoparticle stock solution. After each deposition, we imaged the evaluation array and automatically counted the number of deposited particles using custom Python code (see Methods and SI Note 1). We observed mean particle densities of λ = 1.5 ± 1.3, 3.5 ± 2.7, 5.3 ± 3.9, and 8.6 ± 5.7 particles per SiN_*x*_ opening in the evaluation array after each successive deposition (Table 2, Figure 5B). Mean particle densities scaled linearly with deposition number (*R* ^2^ = 0.982, regression was performed on the four deposition-level mean values, rather than on individual opening counts), confirming that each deposition adds a predictable number of particles.

**Table 2:** Automated Particle Counts by Optical Microscopy^a^

Depositions	λ	σ	σ^2^/λ	*N*_samples	*N*_particles
1	1.50	1.32	1.16	300	453
2	3.45	2.72	2.15	266	920
3	5.34	3.96	2.94	273	1460
4	8.55	5.69	3.80	267	2285

a λ (mean particles per evaluation opening), σ (standard deviation), σ^2^/λ (variance-to-mean ratio), *N*_samples (number of openings analyzed), *N*_particles (total particles counted). All means are statistically different (*p* < 0.001).

**Figure 5:**
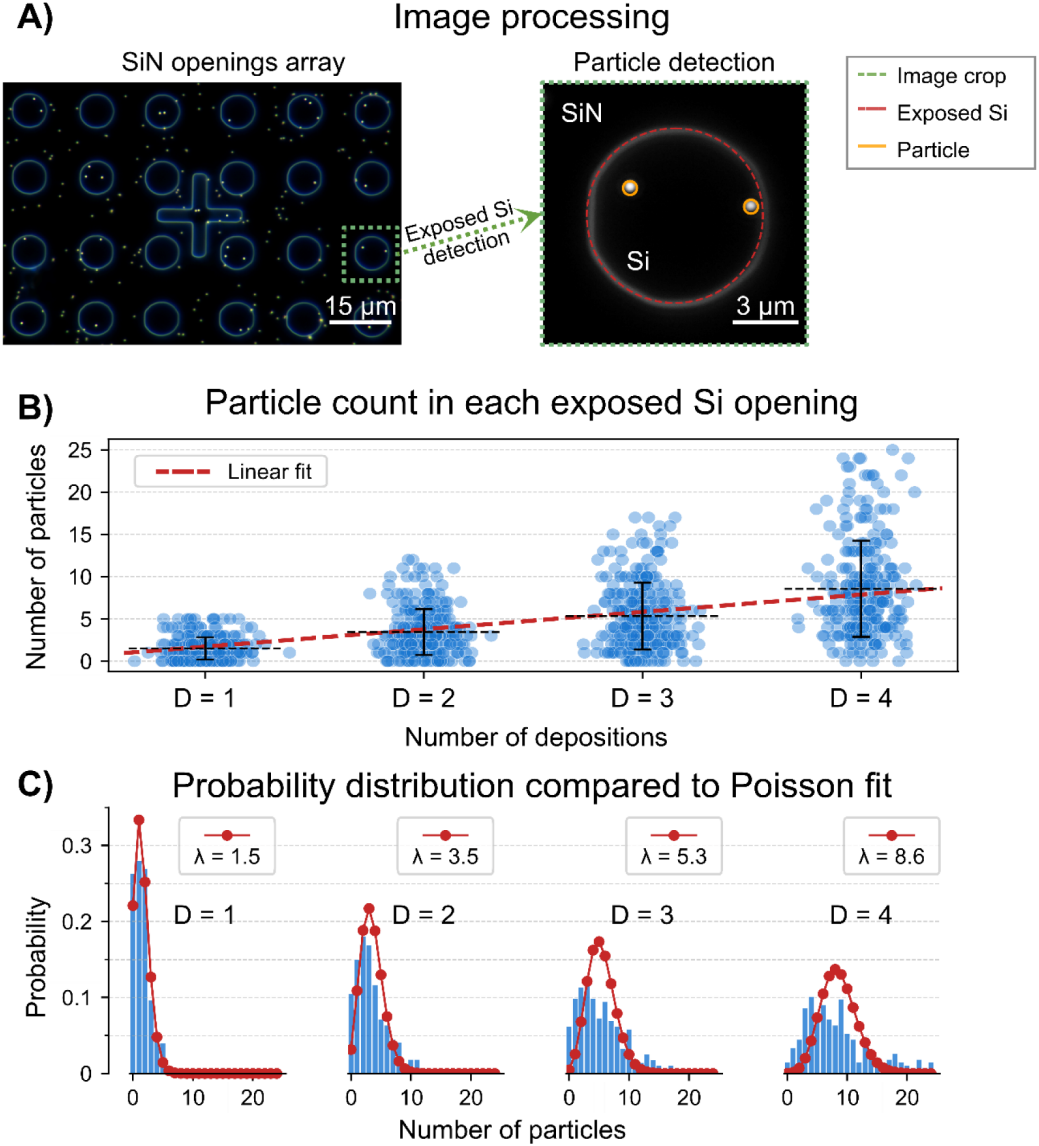
Optical process control for scalable nanopore manufacturing. (A) Left: Epi-dark-field optical microscopy of a portion of one evaluation array showing a 4 × 6 array of 8 μm diameter SiN*_x_* openings. Scale bar: 15 μm. Right: automated detection algorithm identifies each exposed Si area (green dashed box shows one cropped region of interest; red dashed circle defines particle detection area) and counts 200 nm Au particles (yellow circles). Scale bar: 3 μm. (B) Particle deposition density increases linearly with sequential depositions (D1–D4) on the same substrate. Each data point represents one circular 8 μm diameter SiNx opening (∼270 openings per deposition). Black error bars show mean ± SD. Red dashed line: linear fit of the mean values. (C) Particle count distributions (blue histograms) compared to Poisson distributions with matching means (red lines and points; λ = 1.5, 3.5, 5.3, and 8.6 for D1–D4, respectively).

To verify that particle deposition follows Poisson statistics, we compared the observed particle count distributions to theoretically expected Poisson distributions for each deposition number (Figure 5C). At low deposition numbers, we observed close agreement with Poisson behavior, as indicated by variance-to-mean ratios (σ^2^ /λ) near one for D1 and D2 (Table 2). At higher deposition numbers, the distributions showed increasing overdispersion (higher particle counts than expected), with ratios of 2.94 and 3.80 for D3 and D4, respectively. To determine whether this behavior is specific to our deposition conditions or inherent to HF-mediated Au nanoparticle deposition, we reanalyzed particle distribution data from O’Reilly et al.^38^ Although these authors reported visual clustering, they did not perform statistical quantification to assess whether the deposition followed Poisson statistics. Computing σ^2^ /λ from their reported particle counts reveals overdispersion that is similar to our observations (σ^2^ /λ = 1.6–4.8, Supporting Table S1), suggesting that overdispersion at higher particle densities is a general characteristic of HF-mediated citrate-capped Au nanoparticle deposition, rather than an artifact of our specific process conditions.

For practical manufacturing applications, these results indicate that the preferred fabrication strategy is to tailor the particle deposition chemistry (i.e., nanoparticle concentration in solution) to achieve the desired particle density in a single deposition. For single-nanopore applications (where λ ≈ 1 represents ideal conditions), the current protocol using an undiluted nanoparticle stock solution remains well controlled and statistically predictable. A second deposition provides a viable correction strategy when particle deposition counts are lower than desired. However, relying on multiple sequential depositions would compromise process accuracy due to the observed overdispersion at higher subsequent deposition numbers. For applications requiring higher particle densities while maintaining statistical control, increasing the particle concentration in solution, or exposing the Si device layer over a larger area are viable approaches. To confirm that the undiluted Au nanoparticle stock solution provides near-optimal density for single-nanopore applications, we evaluated different water-diluted solutions (12.5%, 25%, and 50% v/v). All dilutions yielded λ ≈ 0.4–0.5 deposited particles per SiN_*x*_ opening, approaching the practical detection limit and being significantly lower than the results when using undiluted stock solution (two-sample z-test, *p* < 0.001). This confirmed that for our device design the undiluted Au nanoparticle stock solution provides the most favorable conditions for achieving a single nanopore per membrane, which typically is most relevant for biosensing applications.

#### 2.2.2 Post-MACE Pore Verification

To demonstrate post-MACE process control of the formed nanopores, we investigated the conditions under which BOX undercuts surrounding nanopore openings were visible under optical microscopy. Energy-filtered TEM (EF-TEM) thickness measurements revealed that undercuts that have a diameter of >180 nm form complete channels through the Si/BOX membrane stack with the full BOX thickness removed beneath the pore, while undercuts that have a diameter of <150 nm retain residual BOX (Figure S2). BOX undercuts of 180 nm or larger are readily visible in differential interference contrast (DIC) bright-field imaging (Figure 1F), making them well-suited for confirming successful pore formation. Because each undercut is centered on a single nanopore, this optical pass/fail signature also distinguishes single- from multiple-pore membranes at the device level, a discrimination that conductance-based feedback used in CBD cannot resolve. The same signature also rules out unaccounted defects: any pinhole or other channel through the Si device layer would itself produce a BOX undercut, so a single concentric undercut around the pore confirms the absence of additional channels in the Si. This establishes a complete two-stage optical process control workflow: (1) count particles deposited on the exposed Si surface before sMACE, to predict pore formation probability (see Section 2.2.1), and (2) inspect devices after the sMACE process for visible undercuts as rapid pass/fail verification of pore formation. Devices that fail either process control stage can be cleaned and returned to the deposition step, limiting material waste and enabling precise control over the resulting nanopore density. To validate this workflow, we characterized 432 membranes by SEM, counting undercuts as a proxy for nanopore formation. Of these, 108 (25%, 95% CI [0.21, 0.29]) contained at least one nanopore, with 68 containing exactly one: the configuration most relevant for single-molecule sensing. Under stochastic deposition conditions, the yield of membranes with precisely one pore is capped to ∼37% (1/e). Thanks to the second process control step, devices with zero pores can either be reprocessed (minimizing waste) or discarded (optimizing throughput).

### 2.3 Electrical Validation

To verify that sMACE pores in *t* = 18 nm membranes are conductive and suitable for sensing, we benchmarked their electrical and noise performance against an established fabrication method. We measured two sMACE devices (one with a single conductive pore and one with 16 conductive pores) alongside two conductance-matched CBD devices, all characterized on the same setup in 1 M KCl buffered with 10 mM HEPES at pH 8.0 (Figure S3, Table S2; see Methods and Supplementary Note 2 for details).

To characterize open-pore conductance, we recorded current–voltage (I–V) sweeps between ± 300 mV. The sMACE devices exhibited linear I–V characteristics with no rectification, whereas the higher-conductance CBD device revealed slight asymmetry (Figure S3A). Applying the cylindrical-pore model^39^ with the 18 nm Si device layer as pore length yielded a per-pore diameter of ∼5 nm for both sMACE devices (single-pore and 16-pore configurations; Table S2), in agreement with the pooled TEM statistics (Table 1). Both values exceed the reported leakage of intact control membranes by more than 2 orders of magnitude.^29^

To quantify the nanopore electrical noise, the root-mean-square current (*I*_rms_) at 300 mV applied bias and the power spectral density (PSD) were recorded for all four devices (Figure S3B, C). The single-pore sMACE device matched the low-conductance CBD reference within measurement precision, and both fell within the range reported in the literature for nanopores of comparable dimensions.^9^ The multipore sMACE device showed proportionally higher noise consistent with 16 parallel conduction pathways.

To confirm DNA-sensing through nanopores formed in *t* = 18 nm membranes, we translocated 2 kbp double-stranded DNA across the multipore sMACE device. We detected 4162 translocation events at 300 mV over a period of 32 min (Figure S3D). Fitting the event density yielded a peak conductance-blockade (ΔG) of 7 ± 1 nS. Across 16 parallel pores, this dispersion is only consistent with comparable individual pore diameters. Conversely, a broad distribution of pore diameters would be expected to produce a broader and potentially more heterogeneous blockade amplitude distribution. Via the cylindrical-pore model, the ∼1 nS spread in ΔG corresponds to ≈0.4 nm pore-to-pore diameter variability, consistent with the pooled TEM statistics (Figure S1, Table 1). Applying the same model to the 7 ± 1 nS blockade yielded effective pore dimensions of *d* = 2.7 ± 0.2 nm and *L* = 3.5 ± 0.7 nm.^39^ The smaller diameter, estimated using the DNA molecular ruler, suggests that the pore diameter tapers as it approaches the Si-BOX interface, resulting in an effective pore length much shorter than the Si device layer thickness. Reduced nanopore length is known to improve the sensor SNR,^9^ and is unlikely to be achieved in the proportional MACE regime, where pore length tracks the full Si device layer thickness.

## 3 Conclusions

We established a scalable fabrication method for realizing 4 nm diameter nanopores in ultrathin Si membranes on SOI substrates that leverages the observation that particles up to 50 times the target pore size can reliably form nanoscale features via sMACE. We identified the particle-to-Si membrane thickness ratio (*d*/*t* ≥ 0.8) as the governing parameter for self-limiting MACE behavior, revealing that particles ranging from 15 to 200 nm all form 4 nm diameter pores. Leveraging this understanding, we designed a fabrication process using 200 nm Au nanoparticles that enables two stages of optical process control: particles deposited on the Si surface are visible before etching, and the resulting pores create optically detectable undercuts after sMACE, providing optical verification at both fabrication stages.

We demonstrated the two-stage process-control workflow, quantified the density of deposited Au nanoparticles, verified pore formation, and confirmed the DNA-sensing capability of single- and multiple-pore sMACE devices, with noise performance comparable to CBD references of comparable conductance. Open-pore conductance yielded a per-pore diameter of ∼5 nm, in agreement with the 4 nm TEM measurement, while DNA conductance-blockade analysis indicated pore diameter tapers near the Si-BOX interface, yielding an effective pore length shorter than the Si device layer thickness, a geometry favorable for sensor signal-to-noise. The narrow conductance-blockade distribution across 16 parallel pores provides independent electrical evidence supporting the narrow pore-diameter population established by TEM.

Our systematic mapping of the sMACE parameter space, including Au particle diameter, Si membrane layer thickness, and solution chemistry, provides a design framework for tailoring the number and dimensions of nanopores. In contrast to serial nanopore-fabrication methods such as TEM drilling and controlled dielectric breakdown, which rely on per-pore feedback during fabrication, sMACE is intrinsically parallel and uses standard wet-chemistry and microscopy equipment. sMACE thus establishes a practical manufacturing platform for realizing 4 nm nanopores in Si nanomembranes, with promising applications in biomedical diagnostics, single-molecule analysis, and DNA sequencing.

## 4 Materials and Methods

The methods described below characterize the sMACE parameter space and validate the two-stage optical process control workflow. Electron microscopy provided quantitative characterization of nanopore dimensions and BOX undercut geometry, confirming the robustness of the proposed optical process control. To characterize particle deposition statistics and their relationship to pore formation, we performed sequential depositions (1–4 times) on the same substrate and quantified the resulting particle densities and nanopore yields. While this study prioritized systematic parameter mapping over yield optimization, the optical inspection protocol demonstrated here operates independently of electron microscopy during production, requiring only standard optical microscopy for particle detection and to confirm successful nanopore formation.

### 4.1 Nanopore Fabrication

#### 4.1.1 Silicon Nanomembrane Fabrication

Nanomembranes were fabricated from ⟨100⟩ SOI wafers (Soitec, France) with a 70 nm thick Si device layer, a 145 nm thick BOX layer, and 400 μm thick Si handle layer, following the process adapted in our previous work.^29^ The Si device layer was thinned to *t* = 5, 12, or 18 nm by thermal oxidation in dry O_2_ at 1000 °C for 300, 270, or 226 min, respectively. A 270 nm thick SiN_*x*_ passivation layer was deposited by PECVD (Precision 5000 Mark II, Applied Materials, USA) on the frontside (the side of the Si device layer) of the SOI wafer. For processing of the backside (the side of the Si handle layer) of the SOI wafer, the frontside SiN_*x*_ was protected with blue tape during a 3 min buffered oxide etch (BOE, 10:1 vol/vol 40% NH_4_F to 49% HF) to remove the thermal SiO_2_ from the handle layer, followed by deposition of a 270 nm thick SiN_*x*_ hard mask by PECVD. Photolithography and reactive ion etching (Precision 5000 Mark II, Applied Materials, USA) were used to pattern both sides: backside openings for membrane release and frontside openings to expose the Si device layer. Anisotropic KOH etching (25% solution, 90 °C) through the backside openings released freestanding 50 μm × 50 μm membranes, with the BOX serving as an etch stop. Each membrane, consisting of the SiN_*x*_ layer on top of the Si device layer and BOX layer below, contained a single 8 μm diameter opening in the SiN_*x*_ layer at its center for nanopore channel formation in the exposed Si device layer, with four additional 6 × 6 arrays of 8 μm SiN_*x*_ openings patterned on adjacent regions beside the suspended membrane for evaluating the particle deposition process. Wafers were diced into substrates of 2.5 mm × 7.5 mm (3 membrane devices) or 5.0 mm × 7.5 mm (6 membrane devices), with one membrane per device.

#### 4.1.2 Au Nanoparticle Deposition

Substrates were cleaned in acetone (10 min) and piranha solution (3:1 vol/vol H_2_SO_4_:H_2_O_2_, 10 min) to remove organic contaminants. Blue tape protected the backside BOX throughout subsequent frontside processing. SiO_2_ was removed from the exposed Si surfaces by drop-casting 10 μL of HF (49%) for 10 s, followed by rinsing in deionized water, ethanol (99%), and nitrogen drying. To systematically investigate the relationship between particle size, Si device layer thickness, and dimensions of the resulting nanopores, citrate-capped Au nanoparticle solutions (Nanopartz Inc., USA; *d* = 10, 15, 20, 40, 100, and 200 nm) were deposited. Stock solutions were stored per manufacturer specifications, with 0.5 mL working aliquots prepared weekly and vortexed (1 min) followed by bath sonication (5 min) before each use to ensure uniform dispersion. For deposition, 20 μL (or 10 μL for 3-devices substrates) of nanoparticle solution was drop-cast onto the substrate surface, immediately followed by drop-casting an equal volume of 5% HF to destabilize the citrate capping of the Au nanoparticles, and to promote Si surface adhesion of the nanoparticles. After 30 s, substrates were rinsed sequentially in deionized water and ethanol (99%), then dried with nitrogen. For studies on the nanoparticle deposition density, diluted solutions (12.5%, 25%, 50% v/v in water), or multiple sequential depositions were employed.

#### 4.1.3 Self-Limiting Metal-Assisted Chemical Etching (MACE)

Nanopores were formed using a MACE solution with molar ratio ρ = [HF]/([HF] + [H_2_O_2_]) = 0.998 (DI:HF:H_2_O_2_ volume ratio of 160:320:1, with HF and H_2_O_2_ concentrations of 49% and 30%, respectively), freshly prepared before each use, unless otherwise noted. This HF-rich solution prevents elongated track formation and instead produces straight, cylindrical pores perpendicular to the ⟨100⟩ Si surface. The high HF concentration also accelerates BOX undercut formation and facilitates particle detachment at the Si-BOX interface, thereby preventing pore enlargement from lateral catalyst motion. With blue tape protecting the backside, 20 μL (or 10 μL for 3-device substrates) of MACE solution was applied to the frontside for 60 s, sufficient to form nanopores with optically visible undercuts while preserving the structural integrity of the BOX layer. Substrates were then rinsed in deionized water and dried with nitrogen after tape removal.

### 4.2 Fabrication Process Control and Validation

#### 4.2.1 Optical Microscopy

Two-stage optical inspection provided process control checkpoints during fabrication. Before sMACE, epi-dark-field illumination enabled nanoparticle counting: SiN_*x*_ openings appeared as blue 8 μm diameter circles, with deposited Au nanoparticles visible as bright spots against the dark background. After sMACE, epi-bright-field microscopy with DIC detected BOX undercuts (200–300 nm diameter) as regions of reduced brightness, confirming successful nanopore formation. This workflow enables manufacturing decisions without the need for electron microscopy or electrical nanopore characterization. Membrane devices with insufficient particle density or without pores can be reprocessed. Images were acquired using an ECLIPSE L200ND microscope (Nikon Corporation, Japan) equipped with Nikon CFI TU Plan APO BD objectives (50×, 100×, 150×), a CMOS color camera (DS-10 RGB, 23.9 Mpix, Nikon), and NIS-Elements Advanced Research software. To characterize particle deposition statistics, images were collected at 150× magnification at five locations per membrane device after each of four sequential depositions and after sMACE, at the membrane device center (0, 0) and at four positions at (0 μm, ± 800 μm) and (±800 μm, 0 μm). Each 6000 × 3984 pixel image (16.1 nm/pixel resolution) captured the 6 × 4 openings of the 6 × 6 evaluation array within the field of view (Figure 1B) and was stored as 16-bit.nd2 files for automated particle counting.

#### 4.2.2 Electron Microscopy

SEM enabled verification of BOX undercut formation during process development. Images were acquired using an Ultra 55 SEM (Zeiss, Germany) at an accelerating voltage of 6 keV and a working distance of 4 mm. For quantitative nanopore geometry characterization, TEM was performed at 200 keV using a 2100F microscope (JEOL Ltd., Japan). Immediately before imaging, samples were treated with oxygen plasma (O_2_/Ar mixture, 5 min, Model 1020, Fischione Instruments) to remove surface carbon contamination. Bright-field TEM images were analyzed using DigitalMicrograph (Gatan Inc., USA) and ImageJ (Rasband, USA). Nanopore diameters were measured by drawing oval selections in ImageJ and calculating the average of the width and height, using images acquired at 0.041 nm/pixel resolution. EF-TEM thickness mapping verified complete removal of the BOX beneath the nanopores. Total intensity (I_t) and zero-loss filtered intensity (I_0) images were acquired with identical settings, corrected for sample drift, and used to calculate relative thickness t/λ = -ln­(I_0/I_t) according to standard procedures.^40^ BOX thickness measurements at the nanopore locations either confirmed fully etched undercuts (appearing as regions approaching zero relative thickness), or they quantified the thickness of the remaining BOX layer.

#### 4.2.3 Image Analysis

Automated particle counting enabled high-throughput quantification of Au nanoparticle deposition across 1 substrate, 3 devices, 12 evaluation arrays, and 288 SiN_*x*_ evaluation areas with exposed Si per deposition condition (24 of 36 openings per array within the optical imaging field of view). Custom Python scripts detected the 8 μm diameter evaluation openings in the SiN_*x*_ layer in each dark-field image using the Hough Circle Transform algorithm (OpenCV), then identified individual Au nanoparticles within each opening based on their characteristic bright appearance against the dark background. Outliers were identified using the interquartile range method and excluded before statistical analysis. Particle counts per evaluation opening were collected to generate particle deposition statistics, and Poisson behavior was validated by computing the variance-to-mean ratio (σ^2^/λ), with values near unity indicating random deposition and values above unity indicating overdispersion. Observed particle distribution densities were also visually compared with theoretical Poisson distributions. Linear regression of mean particle density versus deposition number quantified the incremental particle addition per each subsequent particle deposition cycle. Complete image processing workflows and algorithm parameters are provided in Supplementary Note 1.

#### 4.2.4 Electrical Measurements

Open-pore conductance, noise, and DNA translocation events were recorded with an eNPR Nanopore Reader (Elements SRL, Italy) at a sampling rate of 200 kHz on the 200 nA current range, inside a Faraday cage. Chips were sealed between two silicone gaskets in a custom 3D-printed flow cell (Form 3+, Clear Resin v5, Formlabs), and both reservoirs were filled with 1 M KCl buffered with 10 mM HEPES at pH 8.0 (ionic conductivity 11.8 S/m). Ag/AgCl electrodes provided electrical contact. Current–voltage sweeps were recorded between ±300 mV, and conductance was extracted by linear regression. *I*_rms_ and PSD were computed from recordings at 300 mV applied bias. DNA translocation measurements were performed with 2 kbp double-stranded DNA (UltraPure Calf Thymus DNA Solution, ThermoFisher) at 40 nM, and events were detected and analyzed with EventPro following our previous protocol.^29^ Pore diameter and length estimates were obtained from open-pore conductance and DNA conductance-blockade using the cylindrical-pore model of Charron et al.^39^ Fabrication of the CBD reference devices and full details of the measurement setup are described in Supplementary Note 2.

## Supplementary Material



## Notes

Pooled pore-diameter distribution (Figure S1); automated particle detection methodology and reanalysis of literature data (Supplementary Note 1, Table S1); EF-TEM thickness characterization (Figure S2); electrical characterization of sMACE and CBD nanopore sensors (Supplementary Note 2, Table S2, and Figure S3 (PDF)
