# Supplementary material for: Scalable Fabrication of 4 nm Silicon Nanopores by Self-Limiting Metal-Assisted Chemical Etching Combined with Optical Process Control
Source: Langmuir. 2026 Jun 25;42(26):18788–800. doi: 10.1021/acs.langmuir.6c00975 (PMC13352619; doi:10.1021/acs.langmuir.6c00975)
Supplement: Supplementary file 1 [file la6c00975_si_001.pdf]

Supporting Information

Scalable Fabrication of 4 nm Silicon Nanopores by Self-Limiting Metal-Assisted Chemical Etching Combined with Optical Process Control

*Authors: Fabio De Ferrari[a], Alessandro Enrico[b], Chrysovalantou V. Leva[a], Shyamprasad N. Raja[a], Anna Herland[c,d], Frank Niklaus[a], and Göran Stemme[a]**

Affiliations:

[a]Department of Micro and Nanosystems
KTH Royal Institute of Technology
Malvinas väg 10, 100 44, Stockholm, Sweden

[b]Synthetic Physiology Lab, Department of Civil Engineering and Architecture
University of Pavia
Via Adolfo Ferrata 9, 27100 Pavia, Italy

[c]Division of Nanobiotechnology,
SciLifeLab, Department of Protein Science
KTH Royal Institute of Technology,
Tomtebodavägen 23a, Solna 171 65, Sweden

[d]AIMES - Center for Integrated Medical and Engineering Science,
Department of Neuroscience,
Karolinska Institute, 17177 Stockholm, Sweden.

*Email: stemme@kth.se

Keywords: nanopore sensors, ultrathin membranes, silicon-on-insulator, MACE, self-limiting etching, parallel fabrication, process monitoring

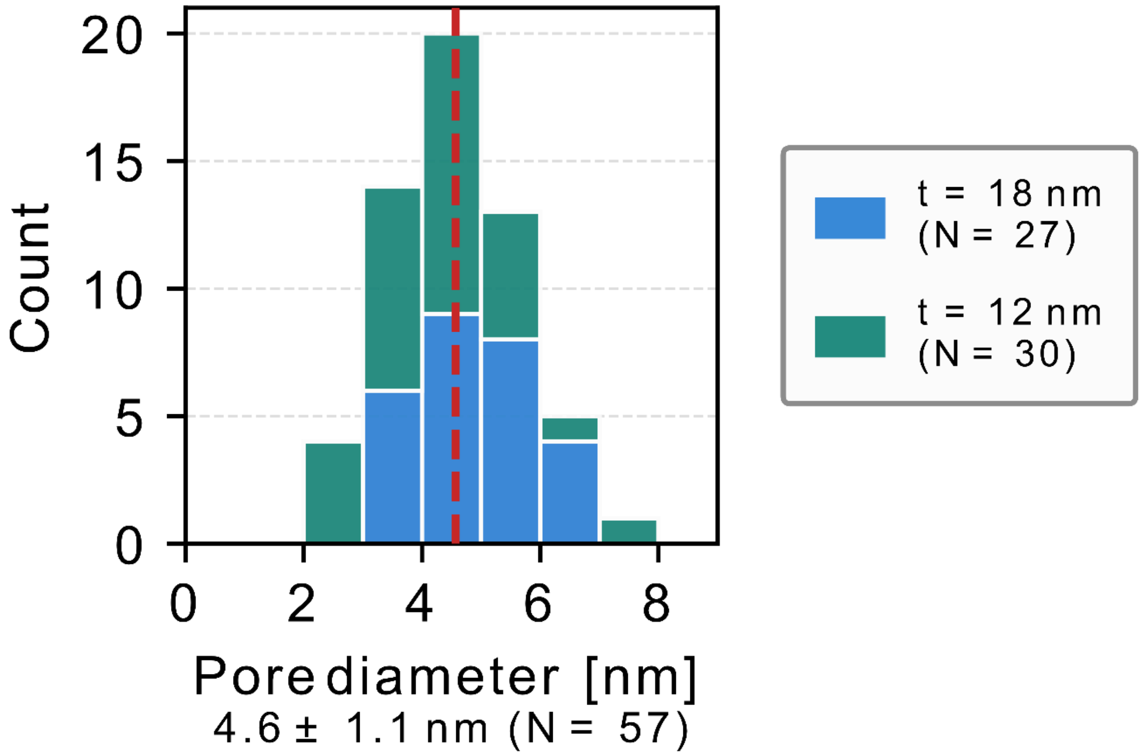

Figure S 1. Pore diameter distribution for all measured pores fabricated under self-limiting conditions ($d/t \geq 0.8$). Blue: t = 18 nm (N = 27), green: t = 12 nm (N = 30). Dashed red line: pooled mean, 4.6 ± 1.1 nm (N = 57).

Table S 1. Particle counts from O'Reilly *et al.*[1]

Depositions	λ	σ	σ^2/λ
1	14.1	5.4	2.0
2	24.7	6.3	1.6
5	41.6	11.5	3.2
10	78.4	19.4	4.8

λ (mean particles per opening), σ (standard deviation), σ^2/λ (variance-to-mean ratio).

Supplementary note 1: automated particle detection and statistical analysis

Automated Au nanoparticle counting was performed using custom Python scripts (Python 3.12.7) with OpenCV (4.12.0), pandas (2.3.3), numpy (2.3.4), scipy (1.16.3), tifffile (2024.12.12), and nd2 (0.10.3) libraries. The analysis pipeline consisted of three stages: (1) Detection of 8 μm diameter openings in SiN_x layer in full-field dark-field images, (2) particle detection within each opening, and (3) statistical analysis with Poisson validation.

All code and representative datasets are available upon reasonable request from the authors.

Detection of circular openings in SiN_x layer. Dark-field optical microscopy images (6000 × 3984 pixels, 16.1 nm/pixel, .nd2 format) were loaded using the nd2 package. Each image contained a 6 × 4 array of test openings, visible as blue 8 μm diameter circles against the dark background (main-text Figure 5(A), left).

Openings were detected using the Hough Circle Transform (OpenCV cv2.HoughCircles with HOUGH_GRADIENT method) with the following parameters: dp = 1, minDist = 620 pixels (~10 μm), param1 = 30, param2 = 20, minRadius = 184 pixels (~3 μm), maxRadius = 310 pixels (~5 μm). Detected circles were validated against expected array positions; missing detections raised an error.

For each circle, a unique identifier was created that encodes: fabrication date (mmdd), number of depositions, device number, array location (0 center, 1-4 for top/bottom/left/right), and position within the array (A-D rows, 1-6 columns). Example: S1119_D2_c2_a1_B5. A pandas DataFrame stored source file metadata, paths, and detected circle size and position.

Image cropping. For each detected opening in the SiN_x layer, a square ROI (852 × 852 pixels) was cropped centered on the circle center. The green channel was extracted (Au nanoparticles scatter at all visible wavelengths; green was selected to minimize file size while avoiding interference from the blue SiN_x edge) and saved as 8-bit TIFF files with circle identifier as filename: S{date}_D{deposition}_c{device}_*{array}*_{position}.tif. This preprocessing reduced the file size from ~140 MB per full-field image to ~1 MB per cropped ROI. Saved file paths were added to the DataFrame.

Particle detection. Cropped images were processed individually to detect Au nanoparticles, which appeared as bright circular regions. Each image was preprocessed with edge blur (Gaussian kernel = 21 pixels) to reduce false positives at the SiN_x edge, then masked to set pixels outside the ROI to zero. Particles were detected using cv2.HoughCircles (HOUGH_GRADIENT) with: dp = 1.2, minDist = 20 pixels, param1 = 50, param2 = 15, minRadius = 11 pixels (~0.18 μm), maxRadius = 29 pixels (~0.47 μm). These detection radii correspond to the apparent scattering signature of the Au nanoparticles in dark-field images, which exceeds the physical particle diameter (200 nm) due to the optical diffraction limit. For each image, particle count was recorded in the DataFrame. Detection overlay images (.png format) were saved for quality assessment (main-text Figure 5(A), right).

Statistical analysis.

Outlier removal: For each deposition (D1-D4), outliers were removed using the IQR method: Computed Q_1 (25th percentile) and Q_3 (75th percentile), defined bounds [Q_1 - 1.5×IQR, Q_3 + 1.5×IQR], and excluded openings outside these bounds. Outliers represented <3 % of data points across all depositions.

Summary statistics: After outlier removal, computed for each deposition: mean particle count (λ), standard deviation (σ), sample size (N_samples), total particles (N_particles), and

variance-to-mean ratio (σ^2/λ). For Poisson distributions, $\sigma^2/\lambda \approx 1.0$; values >1 indicate overdispersion (clustering), values <1 indicate underdispersion.

Poisson distribution validation: Observed count distributions were compared with theoretical Poisson distributions having λ equal to the observed mean (main-text Figure 5(C)). Visual comparison showed good agreement at low deposition numbers (D1-D2) but systematic deviations at higher numbers (D3-D4), consistent with the variance-to-mean ratios reported in main-text Table 2.

Linear regression: Mean particle densities from sequential depositions were fitted using ordinary least squares (scipy.stats.linregress) on the four deposition-level mean values rather than on pooled opening-level counts: $\lambda = 2.3 \times N - 1.0$, $R^2 = 0.982$ (main-text Figure 5(B)), where N is deposition number. This choice reflects the overdispersion observed at higher deposition numbers (D3–D4).

Re-analysis of O'Reilly *et al.* data. To assess whether overdispersion is general to HF-mediated Au nanoparticle deposition, we re-analyzed particle distribution data from O'Reilly *et al.*[1] Mean particle count and standard deviation were extracted from their Figure 6(D). For each reported deposition condition, we computed σ^2/λ (Table S 1). Variance-to-mean ratios ranged from 1.6 to 4.8, with higher values at higher particle densities, mirroring our observations and confirming that overdispersion is characteristic of HF-mediated citrate-capped Au nanoparticle deposition.

Detection accuracy and limitations. Accuracy was assessed by visual inspection of overlay images. At low densities (D1-D2), automated detection showed good agreement with visual inspection. At high densities (D3-D4), particle clustering created ambiguity for both methods. This variability reflects the underlying Au particle deposition process (clustering, non-

independence) rather than algorithmic limitations, as evidenced by systematic trends in σ^2/λ and agreement with manual counting studies.[1]

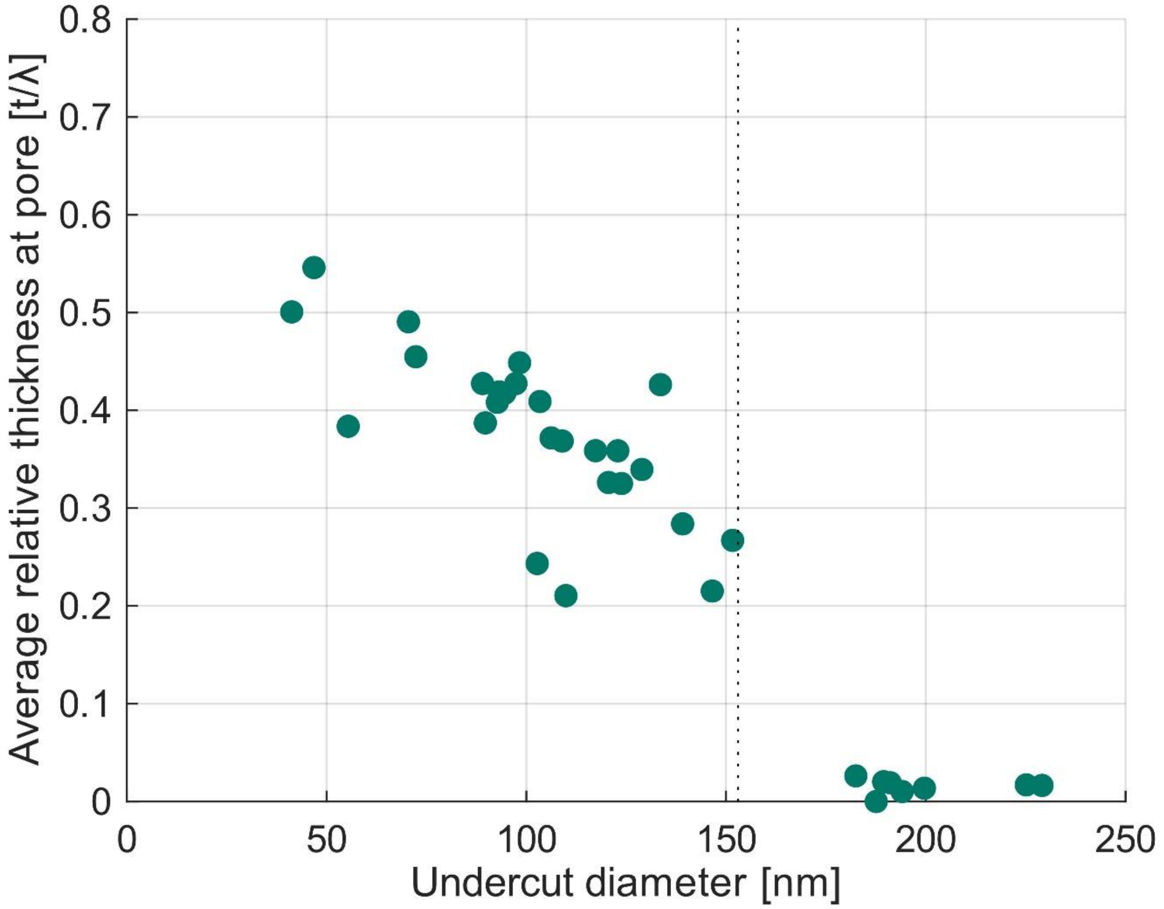

Figure S 2. Energy-filtered TEM (EF-TEM) thickness maps. Measured BOX thickness at the center of each nanopore as a function of undercut diameter. For undercuts smaller than ~150 nm, the BOX thickness decreases linearly with increasing undercut diameter, consistent with isotropic HF etching of the BOX layer from the pore site. For undercuts larger than ~180 nm, the measured thickness approaches zero, indicating that HF etching has penetrated the full BOX thickness and formed an open channel.

Supplementary note 2: electrical characterization of sMACE and CBD nanopore sensors

We previously demonstrated nanopore sensing in 12 nm Si device layers.[2] Because sMACE is a self-limiting process that sets pore diameter independently of device layer thickness, the same sensing capability is expected to carry over to thicker membranes. To confirm this for the 18 nm Si device layer used here, we measured electrical performance, noise, and DNA translocation, benchmarking sMACE against two controlled dielectric breakdown (CBD) devices of comparable conductance fabricated in-house.

To form the CBD pores, we used low-noise NORCADA BDPore SiN_x membranes with a thickness of 20 ± 2 nm. The chip size was 5 × 5 mm^2 and the membrane size was 20 × 20 μm^2. Before fabrication, the chips were cleaned for 5 min in a UV-Ozone cleaner (Jelight Model 30). Each chip was sealed between two silicone gaskets and mounted in a custom 3D-printed flow cell (Form 3+, Formlabs, Clear Resin v5). Both reservoirs were filled with 1 M KCl buffered with 10 mM HEPES at pH 8.0 (ionic conductivity 11.8 S/m). Ag/AgCl electrodes provided electrical contact inside a Faraday cage. The CBD setup, reported previously by other groups,[3,4] consisted of a DC voltage source (Keithley 2220-30-1) for bias, a transimpedance amplifier (DLPCA 200, Femto GmbH), and a DC SMU (Ossila X200) for current measurement, controlled by custom LabView scripts. Nanopores were formed by applying an electric field of 1 V/nm; the time to breakdown at this field was approximately 50–60 s. We selected CBD devices with conductance values matching those of the two sMACE devices (Table S 2). Although the per-device sMACE etch time (60 s) is comparable to the CBD breakdown time per pore (50–60 s), the two are not directly comparable as throughput metrics: sMACE forms all catalyst-defined pores on a substrate in a single parallel etch, while CBD requires sequential breakdown for each individual pore.

Table S 2. Electrical characterization and estimated pore geometry for sMACE and CBD devices measured on the same setup. CBD devices were selected for conductance match to the sMACE devices.

	**sMACE** single-pore	**sMACE** multi-pore	**CBD** small	**CBD** large
Conductive pores [N]	1	~16	1	1
Total conductance G [nS]	11	188	28	247
Per-pore G [nS]	11	~12	28	247
I_{rms} @ 0 mV [pA]	—	162	—	—
I_{rms} @ 300 mV [pA]	187	626	135	178
ΔI_{DNA} @ 300 mV [nA]	—	2.1	—	—
SNR (ΔI_{DNA} / I_{rms})	—	11.2	—	—
PSD @ 10 Hz [pA^2/Hz]	39	407	53	157
Pore diameter [nm] (from open pore conductance)[a]	5.3 ± 0.1	5.6 ± 0.1	9.0 ± 0.4	35.4 ± 1.0
Pore length [nm] (from membrane thickness)	18 ± 1	18 ± 1	20 ± 2	20 ± 2
ΔG_{DNA} @ 300 mV [nS]	—	7 ± 1	—	—
Pore diameter [nm] (from conductance blockade)[a]	—	2.7 ± 0.2	—	—
Pore length [nm] (from conductance blockade)[a]	—	3.5 ± 0.7	—	—
Pore formation time [s]	60	60	50-60	50-60

[a]Pore diameter and length estimated using the model described in Charron *et al.*[5] via the online calculator at www.tcossalab.net/porecalc.

To characterize the electrical and noise performance of both sMACE and CBD devices, we used an eNPR Nanopore Reader (Elements SRL, Italy) with a 200 nA current range. We computed power spectral density (PSD) and root-mean-square current (I_{rms}) from current recordings at a sampling rate of 200 kHz.

Because nanopore noise is setup-dependent,[6] we compared sMACE against CBD by measuring conductance-matched devices on the same setup. We recorded baseline traces for the sMACE and CBD samples at 0 mV, establishing the intrinsic noise floor of the measurement setup. Then recorded I_{rms} at 300 mV for all four devices (Table S 2). The single-pore sMACE device showed I_{rms} comparable to that of the conductance-matched CBD-small device, indicating that sMACE pores perform as well as low-noise CBD pores on this setup. The measured noise is also comparable to values reported for both protein and solid-state nanopores in the literature,[6] supporting the use of this platform for translocation detection.

To further verify the pore diameter, we combined two electrical estimates using the cylindrical-pore model implemented in the online calculator of the Tabard-Cossa group.[5] Taking the 18 nm Si device layer thickness as the pore length, the open-pore conductance yielded diameters of 5.6 and 5.3 nm for the two sMACE devices. Using DNA as a molecular ruler in the multi-pore device, the measured conductance blockade yielded an effective pore diameter of 2.7 ± 0.2 nm and an effective length of 3.5 ± 0.7 nm. This effective diameter is interpretable as a single-pore size only for single-molecule, single-pore events. A larger pore or simultaneous translocations through several pores would broaden the blockade distribution and shift the estimate upward, rather than producing the narrow distribution centered at 2.7 nm that we observe. The low event rate relative to the sub-millisecond dwell times makes simultaneous translocations unlikely. Two implications follow. First, a narrow DNA-blockade distribution is possible only if the 16 constrictions have comparable diameters; a broad diameter distribution across pores would yield a broad blockade histogram. This therefore provides independent evidence that the self-limiting regime produces a narrow-diameter population, consistent with the TEM statistics in the main text. Second, the effective pore length (3.5 nm) is much shorter than the nominal device layer thickness (18 nm), suggesting that the pore narrows from top to bottom, following the particle–membrane interface geometry: a desirable feature of sMACE, since shortened

effective length improves sensing performance by reducing the sampling volume during translocation.[6] Note that the open-pore diameter estimate (~5 nm) is itself an upper bound: it assumes the full 18 nm Si device layer thickness as the pore length, whereas the DNA-derived effective length (3.5 nm) indicates the conductive channel is substantially shorter. A shorter effective length would yield a smaller estimated open-pore diameter, bringing the open-pore conductance-derived value closer to the TEM- and DNA-derived diameters.

The multi-pore device showed 16 undercuts and a total conductance of 188 nS, corresponding to ~12 nS per pore, consistent within precision with 16 equivalent pores each matching the conductance of the single-pore device. We previously verified the absence of leakage through the Si membrane in the absence of nanopores.[2]

To demonstrate that sMACE pores in 18 nm Si support DNA sensing, we recorded translocation events on the sMACE multi-pore device at an applied bias of 300 mV and analyzed them using EventPro, following our earlier protocol.[2] We computed the conductance drop ΔG as the measured current blockade divided by the applied bias. We detected 4162 events over 32 min of recording (Figure S 3(D)), confirming DNA detection capability on devices produced by this fabrication workflow. Figure S 3(E) shows representative translocation events in the unfiltered current recording.

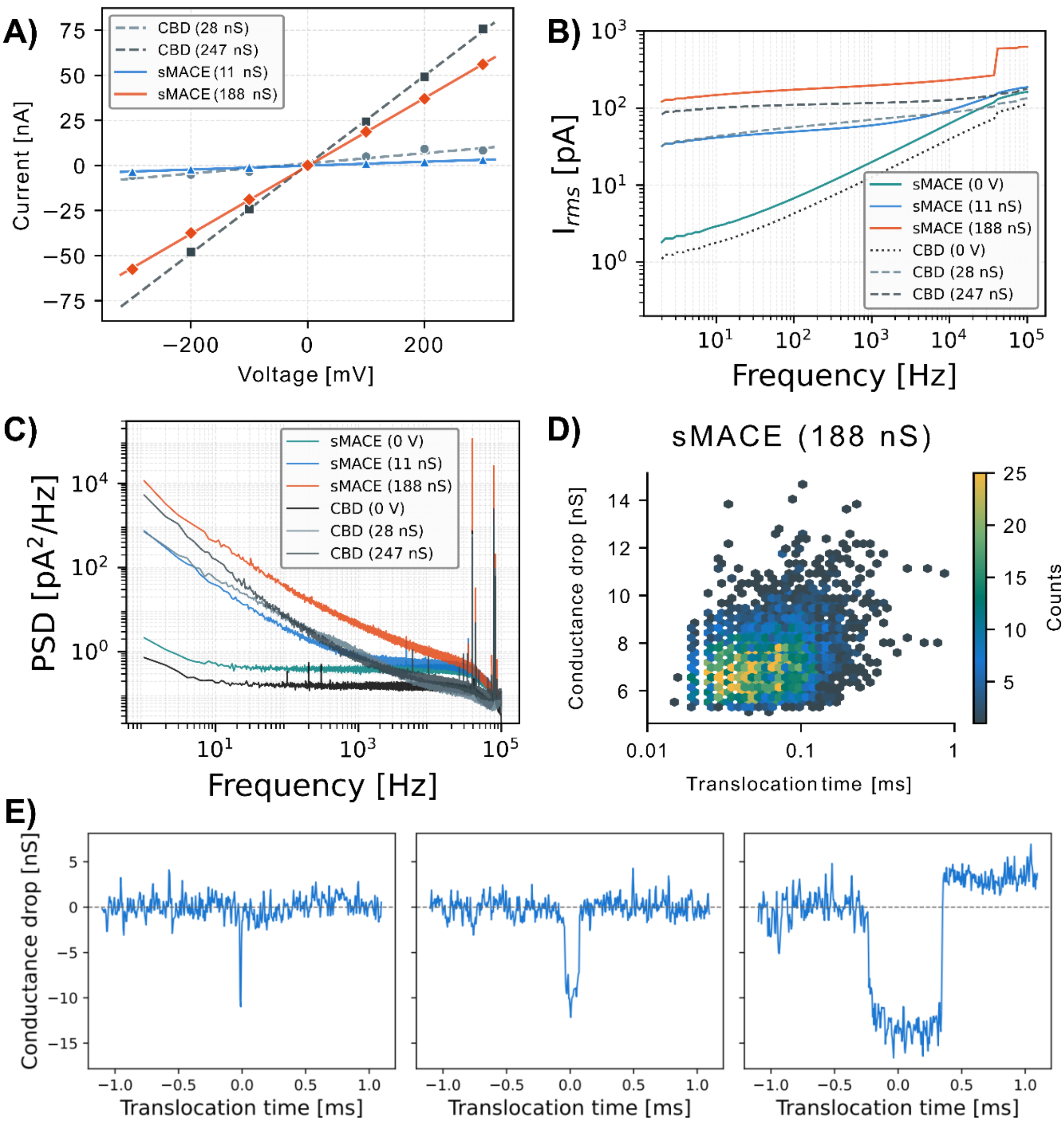

Figure S 3. Electrical characterization of sMACE and CBD nanopores. (A) Current-voltage (I-V) characteristics recorded in 1 M KCl. Linear fits yield the conductances reported in the legend. (B) Root-mean-square (rms) current noise recorded at 300 mV applied bias for sMACE and conductance-matched CBD devices measured on the same setup; 0 mV baseline shows the noise floor. (C) Power spectral density (PSD) of the ionic current recorded under the same conditions as in (B). (D) DNA translocation events (2 kbp dsDNA) through the sMACE 188 nS device at 300 mV applied bias, shown as conductance drop (ΔG) versus translocation time (Δt).

Color scale indicates event counts per bin. (E) Representative conductance-drop traces for translocation events with translocation times of approximately 0.05, 0.1, and 0.8 ms.

Supplementary references

(1) O'Reilly, A. J.; Francis, C.; Quitoriano, N. J. Gold Nanoparticle Deposition on Si by Destabilising Gold Colloid with HF. *J. Colloid Interface Sci.* **2012**, *370* (1), 46–50. https://doi.org/10.1016/j.jcis.2011.12.012.

(2) De Ferrari, F.; Raja, S. N.; Herland, A.; Niklaus, F.; Stemme, G. Sub-5 nm Silicon Nanopore Sensors: Scalable Fabrication via Self-Limiting Metal-Assisted Chemical Etching. *ACS Appl. Mater. Interfaces* **2025**, *17* (6), 9047–9058. https://doi.org/10.1021/acsami.4c19750.

(3) Waugh, M.; Briggs, K.; Gunn, D.; Gibeault, M.; King, S.; Ingram, Q.; Jimenez, A. M.; Berryman, S.; Lomovtsev, D.; Andrzejewski, L.; Tabard-Cossa, V. Solid-State Nanopore Fabrication by Automated Controlled Breakdown. *Nat. Protoc.* **2020**, *15* (1), 122–143. https://doi.org/10.1038/s41596-019-0255-2.

(4) Kwok, H.; Briggs, K.; Tabard-Cossa, V. Nanopore Fabrication by Controlled Dielectric Breakdown. *PLoS One* **2014**, *9* (3), e92880. https://doi.org/10.1371/journal.pone.0092880.

(5) Charron, M.; Roelen, Z.; Wadhwa, D.; Tabard-Cossa, V. Improved Conductance Blockage Modeling of Cylindrical Nanopores, from 2D to Thick Membranes. *Nano Lett.* **2024**, *24* (34), 10527–10533. https://doi.org/10.1021/acs.nanolett.4c02538.

(6) Fragasso, A.; Schmid, S.; Dekker, C. Comparing Current Noise in Biological and Solid-State Nanopores. *ACS Nano* **2020**, *14* (2), 1338–1349. https://doi.org/10.1021/acsnano.9b09353.
